# mHealth Apps Targeting Obesity and Overweight in Young People: App Review and Analysis

**DOI:** 10.2196/37716

**Published:** 2023-01-19

**Authors:** Elena Vlahu-Gjorgievska, Andrea Burazor, Khin Than Win, Vladimir Trajkovik

**Affiliations:** 1 School of Computing and Information Technology University of Wollongong Wollongong Australia; 2 Faculty of Computer Science and Engineering Ss Cyril and Methodius University Skopje Republic of North Macedonia

**Keywords:** behavior change techniques, user interface design patterns, mHealth apps, obesity, lifestyle, mobile app, mobile health, mobile phone

## Abstract

**Background:**

Overweight and obesity have been linked to several serious health problems and medical conditions. With more than a quarter of the young population having weight problems, the impacts of overweight and obesity on this age group are particularly critical. Mobile health (mHealth) apps that support and encourage positive health behaviors have the potential to achieve better health outcomes. These apps represent a unique opportunity for young people (age range 10-24 years), for whom mobile phones are an indispensable part of their everyday living. However, despite the potential of mHealth apps for improved engagement in health interventions, user adherence to these health interventions in the long term is low.

**Objective:**

The aims of this research were to (1) review and analyze mHealth apps targeting obesity and overweight and (2) propose guidelines for the inclusion of user interface design patterns (UIDPs) in the development of mHealth apps for obese young people that maximizes the impact and retention of behavior change techniques (BCTs).

**Methods:**

A search for apps was conducted in Google Play Store using the following search string: [“best weight loss app for obese teens 2020”] OR [“obesity applications for teens”] OR [“popular weight loss applications”]. The most popular apps available in both Google Play and Apple App Store that fulfilled the requirements within the inclusion criteria were selected for further analysis. The designs of 17 mHealth apps were analyzed for the inclusion of BCTs supported by various UIDPs. Based on the results of the analysis, BCT-UI design guidelines were developed. The usability of the guidelines was presented using a prototype app.

**Results:**

The results of our analysis showed that only half of the BCTs are implemented in the reviewed apps, with a subset of those BCTs being supported by UIDPs. Based on these findings, we propose design guidelines that associate the BCTs with UIDPs. The focus of our guidelines is the implementation of BCTs using design patterns that are impactful for the young people demographics. The UIDPs are classified into 6 categories, with each BCT having one or more design patterns appropriate for its implementation. The applicability of the proposed guidelines is presented by mock-ups of the mHealth app “Morphe,” intended for young people (age range 10-24 years). The presented use cases showcase the 5 main functionalities of Morphe: learn, challenge, statistics, social interaction, and settings.

**Conclusions:**

The app analysis results showed that the implementation of BCTs using UIDPs is underutilized. The purposed guidelines will help developers in designing mHealth apps for young people that are easy to use and support behavior change. Future steps involve the development and deployment of the Morphe app and the validation of its usability and effectiveness.

## Introduction

Obesity has nearly tripled in the last 30 years, with the World Health Organization estimating that around 340 million or 27% of the world’s children and adolescents are overweight or obese [1]. Overweight and obesity have been linked to several serious health problems and medical conditions, including an increase in the risk for noncommunicable diseases such as cardiovascular diseases, diabetes, musculoskeletal disorders, endometrial cancers as well as other types of cancers [1]. Excessive weight and obesity can lead to not only physiological medical complications but also severe psychological effects [2]. The social and emotional well-being and self-esteem of young people are especially impacted during this important developmental phase of life, with these negative consequences tracking well into an individual’s later life [3,4]. Further, there is a general reduction in the intake of certain food groups and nutrients and an increase in the consumption of junk food and sugary drinks [5,6], as well as a significant decrease in engagement in moderate-to-vigorous physical exercises during this transition period between adolescence and adulthood [7]. Therefore, targeting young people (age range 10-24 years) is very important [8].

The assumption that nutrition and physical activity behaviors are mediators of body weight provides the basis for behavioral interventions for obesity, which are largely derived from the principles of classical conditioning and social theories [9]. A person’s behavior is predominantly responsible for maintaining health and plays an important role in the prevention, management, and treatment of overweight and obesity. Behavior change techniques (BCTs) are descriptors (replicable components of an intervention) designed to enable behavior change by addressing important targets of capability, opportunity, and motivation. The refined taxonomy of BCT—Coventry, Aberdeen, and London-Refined (CALO-RE)—is specifically tailored toward the change of physical activity and promotion of healthy eating behaviors [10].

Mobile phone ownership is ubiquitous, especially among young people. Based on the media use report, 91% of youth between 12 and 15 years of age own a mobile device [11]. The mobile devices are carried by their owners most of the time and are rarely switched off [12]; therefore, they can provide notifications to the users at particular moments, thereby enhancing the engagement and adoption of certain behaviors. These devices can also be used for collecting and analyzing user data, which facilitate the capability to automate certain processes, consequently reducing a user’s cognitive load in navigation and selection activities [13]. These characteristics make mobile phones good candidates for delivering digitally supported obesity interventions.

Mobile health (mHealth) apps present a unique opportunity, particularly for young people, to revolutionize the way health behavior change interventions are delivered [14]. However, despite the potential for improved engagement in long-term interventions [15], health interventions delivered by these devices are short-lived. Literature shows that most users cease mHealth app activity within a few uses, and a quarter of mHealth apps are found to be used only 1 time after installation [16]. The factors that impact the adoption of mHealth apps are well-researched, and there is no significant evidence to suggest that adoption alone can improve an individual’s health [16]. The continuation of use where technology supports user engagement in behavior change is the area that can enhance positive outcomes [15]. Thus, the continuation of use of mHealth apps greatly impacts their overall efficacy and potential for success.

The user interface and experience of mobile apps strongly influence users’ perception and satisfaction and have a strong impact on the app’s adoption and continuation of use [17]. The user interface design patterns (UIDPs) are descriptions of the best practices within user interface design. They are general reusable solutions to commonly occurring problems and can ensure that user interfaces flow well and are easy and enjoyable to use. In addition, UIDPs can reduce the cognitive load and improve the overall performance of the app. Furthermore, literature [18] suggests that the overall “look and feel” of apps impacts the adoption by young people, while the perception that health apps were designed primarily for adults was found to be a barrier in using the app. In this context, applying well-known user interface design principles and patterns [19] can improve the efficacy of mHealth apps and contribute toward its continuation of use.

Previous research in this field is primarily focused on mHealth interventions for the adult population without a specific view of the young people demographics [15]. However, evidence suggests that mHealth interventions may be viable in effecting positive health changes in young people as well [20]. The variable results for using mHealth apps by young people could be also explained by the lack of available apps specifically tailored to offer weight management for this group [14]. Additionally, there is a scarcity of research on the impact of UIDPs on the efficacy of BCTs in mHealth apps for obese young people.

The aims of this research were to (1) review and analyze mHealth apps targeting obesity and overweight and (2) propose guidelines for the inclusion of UIDPs in the development of mHealth apps for obese young people, which maximizes the impact and retention of BCTs.

## Methods

**Figure 1:**
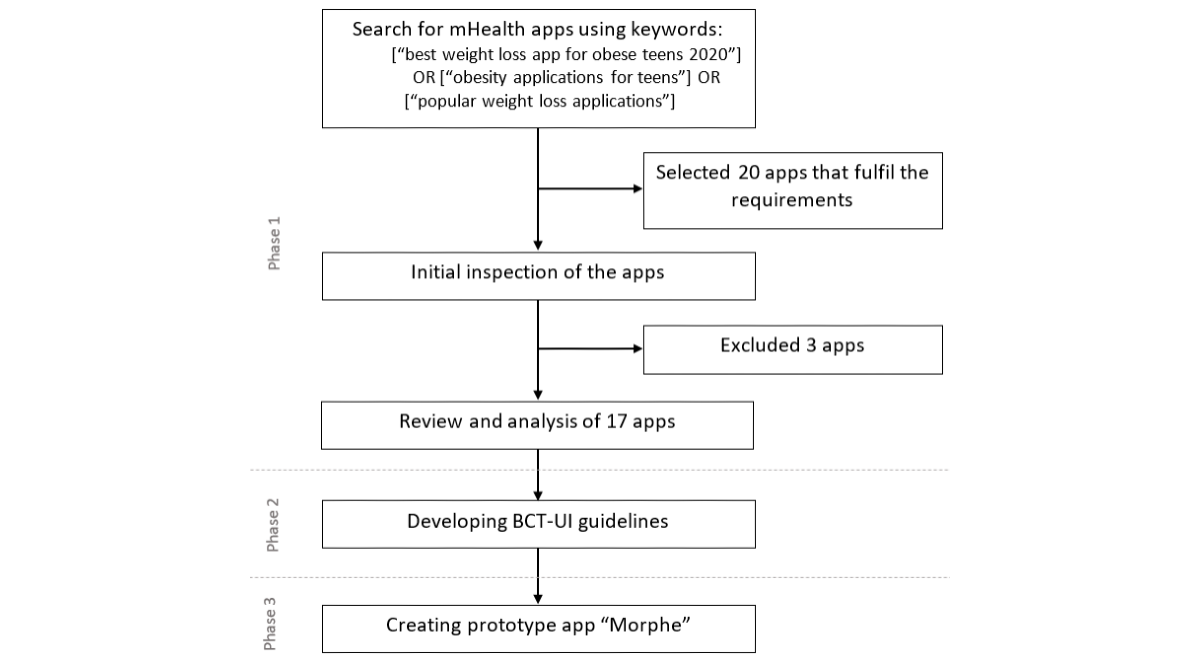
Study methodology diagram. BCT-UI: behavior change technique–user interface; mHealth: mobile health.

The overview of the study methodology is presented in Figure 1. A search for apps was conducted in Google Play Store by using a combination of the following keywords: [“best weight loss app for obese teens 2020”] OR [“obesity applications for teens”] OR [“popular weight loss applications”]. The inclusion criteria for the apps were as follows: free; available in both Google Play and Apple App Store; do not require the use of external devices; appropriate for individuals between 10 and 24 years of age; and app’s primary purpose (app category, tags, and description) is stated as health, nutrition, physical activity improvement, targeting obesity, or specified as a tool for obesity intervention.

The first 20 most popular apps that met the above described criteria were selected for further analysis. During the app inspection, 3 of the selected apps were found to require in-app purchases to access the key functionality and thus were excluded from the study, leaving 17 apps for analysis. Two researchers installed the apps on separate devices and analyzed the features of the apps independently. When there was a discrepancy in the opinions, all the authors discussed them until a consensus was reached.

The selected mHealth apps were analyzed for the inclusion of BCTs from the CALO-RE taxonomy [10] (Multimedia Appendix 1) in their design, taking into account the UIDPs (selected from [19] and [21] Multimedia Appendix 2) used to support those BCTs. UIDPs were noted only in cases where utilized to implement BCTs or some other key functionalities of the app. The generalist UIDPs such as affordance for tap or swipe or key input patterns were not considered in this analysis. However, any glaring problems with an app’s interface that had the potential to disrupt the user’s experience were noted. Additionally, any features that were not available for free (but only as in-app purchases) were not considered in the analysis.

Based on the results of the review and analysis of the apps, BCT-user interface (BCT-UI) design guidelines classifying UIDPs into 6 categories were developed. Furthermore, using the proposed guidelines, a prototype app called “Morphe” was designed. The purpose of Morphe was to showcase the applicability of the BCT-UI guidelines in the development of mHealth apps targeting young people (age range 10-24 years) with obesity.

### Ethical Considerations

As this study does not include experiments on human subjects, no ethical approval was sought.

## Results

### Review of Apps

Based on the app category, descriptions, and offered features, selected apps were classified into 2 groups: (1) apps focused on physical activity (n=12) and (2) apps focused on nutrition (n=5). Our results (shown in the tables below) and Multimedia Appendix 3 indicate that 20 (out of 40) BCTs listed in the CALO-RE taxonomy were present to some degree in the analyzed apps. The most frequently employed BCTs were related to self-monitoring of behavior (15 apps), followed by prompt practice (9 apps), providing feedback on the performance of the behavior (9 apps), goal setting (behavior) (8 apps), and successful behavior contingent rewards (8 apps). The goal setting of behavior and self-monitoring of behavior were mostly implemented in combination (8 apps) as well as by self-monitoring of behavior with prompt practice (9 apps). The goal setting of behavior and goal setting of behavioral outcome were combined in 5 apps. Six of the BCTs implemented in the apps focused on physical activities were not implemented in the nutrition-focused apps (Table 1).

**Table 1:** Number of apps implementing each behavior change technique or user interface design pattern.

Characteristics			Apps (n)
**Behavior change technique^a^**			
	#16: Self-monitoring of behavior	15	
	#19: Provide feedback on performance	9	
	#26: Prompt practice	9	
	#5: Goal setting (behavior)	8	
	#13: Successful behavior contingent rewards	8	
	#6: Goal setting (outcome)	7	
	#17: Self-monitoring of behavioral outcome	7	
	#21: Instruction on how to perform behavior	7^b^	
	#1: Information provision (general)	6	
	#28: Facilitate social comparison	6	
	#22: Demonstrate behavior	6^b^	
	#9: Setting graded tasks	5^b^	
	#12: Effort or progress contingent rewards	4	
	#14: Shaping	4	
	#29: Plan social support	4	
	#40: Stimulate anticipation of future rewards	4	
	#4: Information provision (others’ behavior)	3	
	#3: Information provision (others’ approval)	1^b^	
	#7: Action planning	1^b^	
	#36: Stress management	1^b^	
**User interface design pattern**			
	Content: Cards	14	
	Charts: Sparklines	13	
	Content: Filters	11	
	Gamification-Rewards: Collectibles	10	
	Notifications: Triggers	10	
	Charts: Drilldown	9	
	Content: Search	8	
	Charts: Dashboard	5	
	Form: Calculator	5	
	Social: Profile	5	
	Content: Article list	5^b^	
	Gamification-Rewards: Praise	5^b^	
	Social: Connecting	5^b^	
	Charts: Threshold	4	
	Social: Activity Stream	4	
	Form: Multistep	3	
	Social: Comments	3	
	Social: Groups	3	
	Gamification-Rewards: Points	3^b^	
	Gamification-Rewards: Unlock features	3^b^	
	Gamification: Leaderboard	3^b^	
	Content: Favorites	2	
	Form: Registration with Personalization	2	
	Personalization	2	
	Gamification: Levels	2^b^	
	Social: Reactions	2^b^	
	Charts: Overview plus Data	1^b^	
	Gamification: Appropriate Challenge	1^b^	
	Onboarding: Tutorials	1^b^	

^a^Behavior change techniques are listed as per the numbering in the Coventry, Aberdeen, and London-Refined taxonomy.

^b^Behavior change techniques and user interface design patterns not implemented in apps focused on nutrition.

Our analysis identified several UIDPs used in the implementation of BCTs (Table 1 and Table 2). Information provision was often implemented using cards (14 apps) and complemented with search and filter functionalities. Pattern filters was implemented in 11 apps, and search was included in 8 apps. Favorites was underutilized (only in 2 apps) besides the many obvious opportunities for its implementation. For successful behavior contingent rewards or effort or progress contingent rewards, most apps utilized collectibles (10 apps). Providing feedback was mostly implemented using charts, with sparklines (13 apps) and drilldown (9 apps) included in apps to provide feedback about the performance of the behavior. None of the apps implemented UIDPs such as scarcity, social proof, Kairos, and interactive preview, while tutorials (1 app) and personalization (2 apps) were underutilized. It needs to be noted that 11 of the UIDPs implemented in the apps focused on physical activities were not implemented in the nutrition-focused apps (Table 1).

**Table 2:** Overall characteristics of the apps in this review.

Focus, app name		Type/target group		Behavior change techniques		Total		User interface design patterns
**Physical activity focus**								
	NFL Play 60	Type: Exergaming Educational target group: age range 6-8/9-12 years	#1-Information provision (general) #9-Set graded tasks #12-Effort or progress contingent rewards #13-Successful behavior contingent rewards #14-Shaping		5		Onboarding: Tutorials Content: Cards Charts: Sparklines Gamification–Rewards: Collectibles Gamification–Rewards: unlock features Gamification–Rewards: Points Gamification–Rewards: Praise Triggers Gamification: Levels	
	Runtastic (Adidas Running)	Type: Fitness/physical activity tracking Educational target group: General	#1-Information provision (general) #3-Information provision (others’ approval) #5-Goal setting (behavior) #16-Self-monitoring of behavior #28-Facilitate social comparison		5		Content: Filters Content: Cards Content: Search Form: Multistep Form: Calculator Form: Registration with Personalization Social: Connecting Social: Profile Social: Activity Stream Social: Groups Gamification: Leaderboard	
	7-Minute Workout	Type: Physical activity Target group: General	#13-Successful behavior contingent rewards #16-Self-monitoring of behavior #17-Self-monitoring of behavioral outcome #19-Provide feedback on performance #22-Demonstrate behavior #26-Prompt practice		6		Charts: Overview plus Data Charts: Threshold Content: Filters Content: Cards Form: Calculator Gamification-Rewards: Collectibles Gamification–Rewards: Praise Triggers	
	Sworkit	Type: Fitness/physical activity tracking Educational target group: General, children aged 12 years and younger	#9-Set graded tasks #16-Self-monitoring of behavior #21-Instruction on how to perform behavior #22-Demonstrate behavior #26-Prompt practice		5		Charts: Sparklines Content: Cards Content: Search Content: Filters Content: Article list Triggers	
	Endomondo	Type: Fitness/physical activity tracking Educational target group: General	#5-Goal setting (behavior) #12-Effort or progress contingent rewards #16-Self-monitoring of behavior #19-Provide feedback on performance #28-Facilitate social comparison #29-Plan social support		6		Charts: Sparklines Charts: Drilldown Content: Cards Content: Filters Gamification–Reward: Praise Gamification-Reward: Collectibles Gamification: Leaderboard Social: Connecting Social: Profile Social: Comments Social: Reactions Social: Activity stream	
	Couch to 5K (C25K)	Type: Fitness training Target group: General	#9-Setting graded tasks, #16-Self-monitoring of behavior #21-Instruction on how to perform behavior #26-Prompt practice		4		Trigger	
	Fitify	Type: Fitness/physical activity tracking Educational target group: General	#13-Successful behavior contingent rewards #14–Shaping #16-Self-monitoring of behavior #19-Provide feedback on performance #21-Instruction on how to perform behavior #22-Demonstrate behavior #26-Prompt practice		7		Content: Cards Content: Article list Content: Filters Content: Search Charts: Sparklines Charts: Drilldown Charts: Dashboard Gamification-Reward: Praise Gamification-Reward: Collectibles Triggers	
	Fitness Buddy	Type: Fitness/physical activity tracking Calorie tracking Target group: General	#5-Goal setting (behavior) #6-Goal setting (outcome) #16-Self-monitoring of behavior #17-Self-monitoring of behavioral outcome #19-Provide feedback on performance #21-Instruction on how to perform behavior #22-Demonstrate behavior		7		Content: Filters Content: Search Content: Cards Content: Article list Charts: Drilldown Charts: Sparklines Form: Calculator Form: Multistep	
	FitOn	Type: Fitness/physical activity tracking Educational target group: General	#1-Information provision (general) #4-Information provision (others’ behavior) #5-Goal setting (behavior) #6-Goal setting (outcome) #7-Action Planning #13-Successful behavior contingent rewards #16-Self-monitoring of behavior #21-Instruction on how to perform behavior #22-Demonstrate behavior #26-Prompt practice #28-Facilitate social comparison #29-Plan social support #40-Stimulate anticipation of future rewards		13		Content: Search Content: Filters Content: Article List Content: Cards Content: Favorites Social: Connecting Social: Profile Gamification-Reward: Collectibles Gamification-Reward: Praise Triggers Personalization	
	FitBit	Type: Fitness/physical activity tracking Educational target group: General	#1-Information provision (general) #4-Information provision (others’ behavior) #5-Goal setting (behavior) #6-Goal setting (outcome) #12-Effort or progress contingent rewards #13-Successful behavior contingent rewards #16-Self-monitoring of behavior #17-Self-monitoring of behavioral outcome #19-Provide feedback on performance #21-Instruction on how to perform behavior #22-Demonstrate behavior #28-Facilitate social comparison #29-Plan social support #36-Stress management #40-Stimulate anticipation of future rewards		15		Content: Cards Content: Filters Charts: Dashboard Charts: Sparklines Charts: Drilldown Charts: Threshold Social: Activity Stream Social: Comments Social: Reactions Social: Groups Social: Connecting Social: Profile Gamification: Leaderboard Gamification-Rewards: Collectibles Triggers	
	Zombies, Run!	Type: Exergaming Physical activity Target group: General	#9-Setting graded tasks #13-Successful behavior contingent rewards #14–Shaping #19-Provide feedback on performance #21-Instruction on how to perform behavior #40-Simulate anticipation of future rewards		6		Content: Cards Content: Article List Charts: Sparklines Gamification-Rewards: Collectibles Gamification-Rewards: Unlock features Gamification–Rewards: Points Gamification: Appropriate Challenge	
	Walkr	Type: Exergaming Physical activity Target group: General, kid-friendly	#9-Setting graded tasks #13-Successful behavior contingent rewards #16-Self-monitoring of behavior #26-Prompt practice #28-Facilitate social comparison		5		Gamification-Reward: Collectibles Gamification-Reward: Points Gamification-Reward: Unlock features Gamification: Levels Charts: Sparklines Social: Connecting Triggers	
**Nutrition focus**								
	Cron-O-Meter	Type: Calorie/nutrition tracker Target group: General	#5-Goal setting (behavior) #6-Goal setting (outcome) #16-Self-monitoring of behavior #17-Self-monitoring of behavioral outcome #19-Provide feedback on performance		5		Chart: Dashboard Chart: Sparklines Chart: Drilldown Charts: Threshold Content: Search Content: Filters Content: Cards Form: Registration with Personalization Form: Multistep Form: Calculator	
	Eat the Rainbow Food Journal	Type: Food journal Educational target group: General	#1-Information provision (general) #5-Goal setting (behavior) #12-Effort or progress contingent rewards #14–Shaping #16-Self-monitoring of behavior #40-Stimulate anticipation of future rewards		6		Chart: Drilldown Charts: Sparklines Charts: Dashboard Content: Cards Content: Filters Content: Search Content: Favorites Gamification-Reward: Collectibles Personalization	
	MyFitnessPal	Type: Calorie/nutrition tracker Physical activity tracker Target group: General	#1-Information provision (general) #4-Information provision (others’ behavior) #5-Goal setting (behavior) #6-Goal setting (outcome) #16-Self-monitoring of behavior #17-Self-monitoring of behavioral outcome #19-Provide feedback on performance #26-Prompt practice		8		Content: Cards Forms: Calculator Charts: Sparklines Charts: Drilldown Triggers	
	Fitatu	Type: Food tracker Weight tracker Target group: General	#6-Goal setting (outcome) #16-Self-monitoring of behavior #17-Self-monitoring of behavioral outcome #26-Prompt practice		5		Charts: Sparklines Charts: Drilldown Content: Search Content: Filters	
	Lose It! Calorie Counter	Type: Calorie/nutrition tracker Physical activity tracker Target group: General	#6-Goal setting (outcome) #13-Successful behavior contingent rewards #16-Self-monitoring of behavior #17-Self-monitoring of behavioral outcome #19-Provide feedback on performance #26-Prompt practice #28-Facilitate social comparison #29-Plan social support		8		Content: Cards Chart: Sparklines Chart: Threshold Chart: Dashboard Chart: Drilldown Social: Comments Social: Activity Stream Social: Groups Social: Profile Triggers Gamification-Rewards: Collectibles	

### BCT-UI Design Guidelines

**Figure 2:**
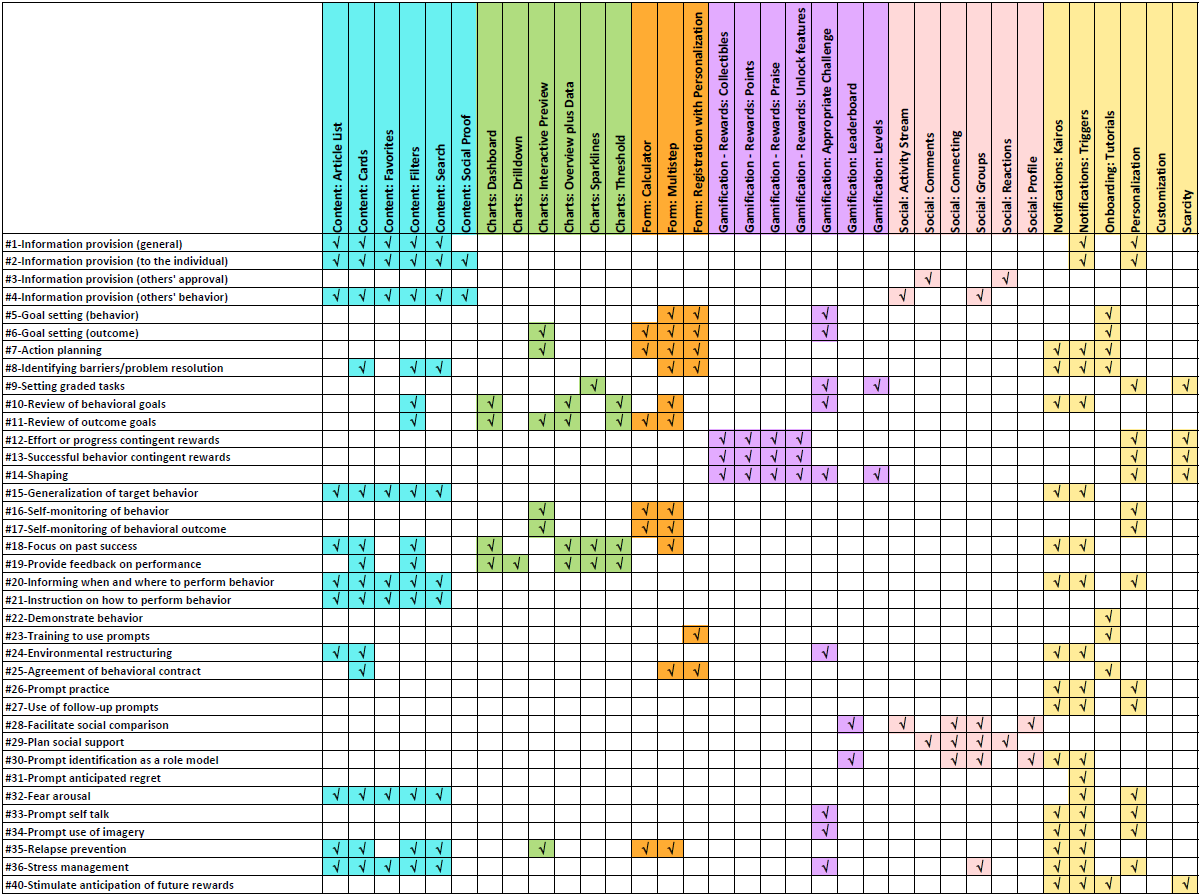
Proposed design guidelines for the use of each user interface pattern in the context of the behavior change techniques.

The app analysis results clearly show that implementing BCTs by using UIDPs is underutilized. Only 12 BCTs and 13 UIDPs are implemented in 5 or more apps, of which 3 BCTs and 3 UIDPs are not implemented in the nutrition-focused apps. To overcome this gap and help developers create mHealth apps for obese young people that are easy to use and effective in motivating users to engage in behavior change, we propose BCT-UI design guidelines (presented in Figure 2). These guidelines focus on implementing BCTs using UIDPs that are impactful for the young people demographics. The guidelines use the BCTs from Michie’s CALO-RE framework [10] with selected UIDPs [19,21,22].

**Figure 3:**
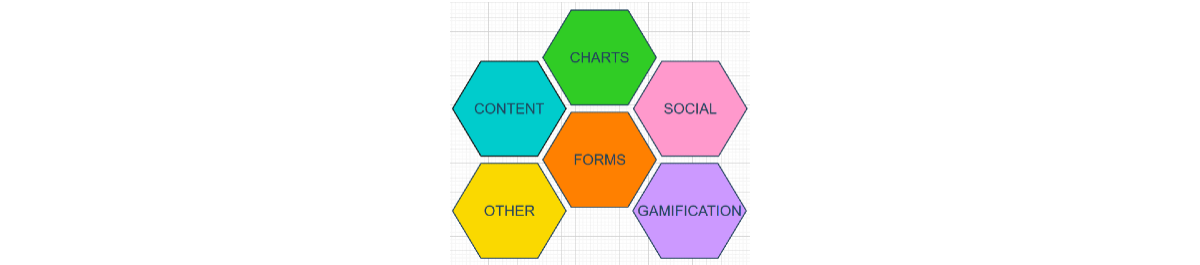
User interface design pattern categories.

Each BCT may have one or more UIDPs appropriate for its implementation. UIDPs are classified into 6 categories (Figure 3). The content represents any display of the information, where the information can be textual or graphical. The presentation of the content can include patterns such as article list, cards, option to mark items as favorites, filter and search to refine or locate content of interest, as well as social proof (that can be presented by textual references). The charts represent how the data can be visually presented, including different types of charts such as dashboard, drilldown, interactive preview, overview plus data, sparklines, or threshold. These user interface patterns can help in visualizing users’ behavioral and outcome goals, action planning, self-monitoring, or present feedback on the performance. The forms category represents umbrella patterns that focus on structure or feature rather than a specific form implementation. In this context, the forms are components that support input from the user. The gamification elements represent the items that deliver rewards or stimulate challenge and competition. In these UIDPs, the rewards can be implemented as collectibles, points, praise, or unlocking specific features and can be used to introduce different challenge levels or create appropriate challenges based on user preferences. The leaderboards as well can be used to facilitate social comparison. The social elements are patterns (activity stream, groups, comments, reactions) that allow users to connect with their peers for social support and comparison. The connection can be anonymous or delivered through social media integration. The “other” category includes patterns that can support delivering prompts of various kinds (such as Kairos and Triggers), tutorials, and general design considerations such as the incorporation of personalization and customization. These UIDPs can be used as nudges to intervene at specific times when the user will be open to receiving advice or performing the goal behavior.

### Use Cases: Using UIDPs to Implement BCTs

The proposed design guidelines offer the utilization of different UIDPs in the implementation of various BCTs. Besides the use cases presented in this section, miscellaneous examples of each pattern in the context of their BCTs are presented in Multimedia Appendix 4. In the given use cases, we have used mock-ups of the Morphe app developed by the authors to showcase the applicability of the proposed BCT-UI guidelines. This app is intended for young people (age 10-24 years) and has 5 main functionalities: learn, challenge, statistics, social interaction, and (user’s) settings. Additionally, the Morphe app uses notifications to nudge the user toward the desired behavior.

#### Use Case 1: Content Presentation

**Figure 4:**
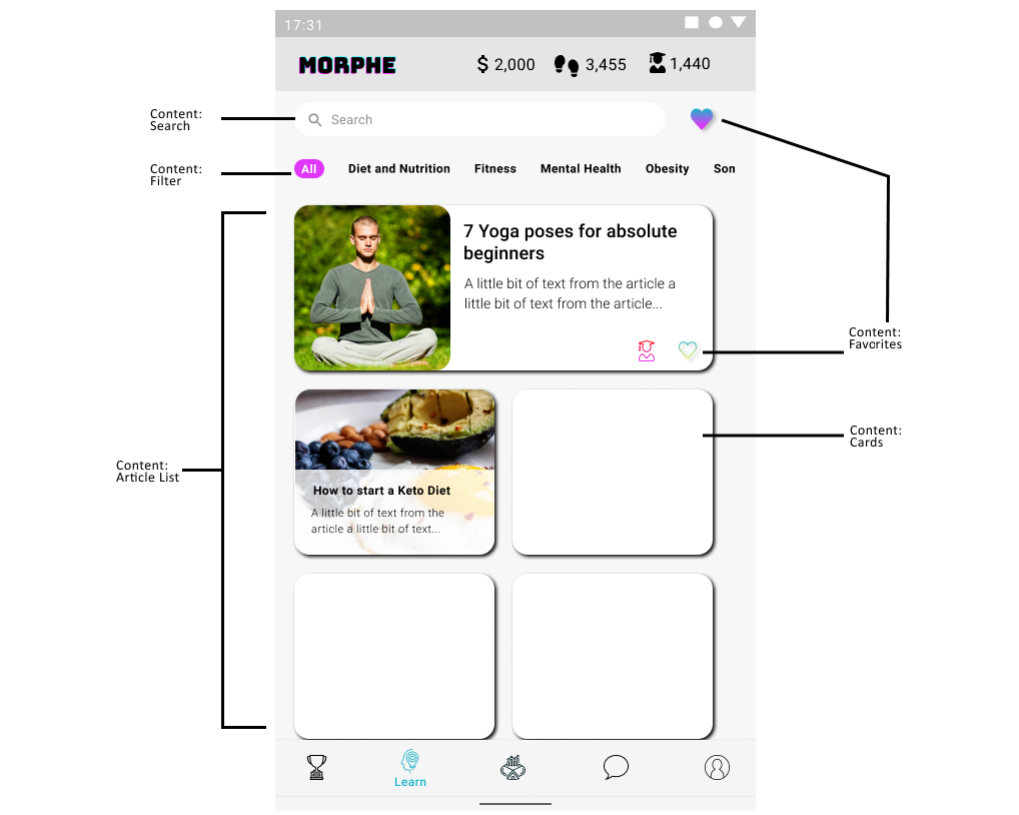
Morphe app educational content.

The presentation of the content in the app is very important for the usability of the app. Content UIDP dictates how app users can access, refine, and interact with app information and experiences. In our Morphe app (Figure 4), the educational content in the “Learn” functionality is presented using the article list pattern. In general, article lists tend to contain multiple rectangular cards used to store and deliver content. Each card contains an image, a title, and a brief description to allow the user to understand what information is contained in the article. Using the favorites pattern, the user can mark the items of interest and have easier access to those articles. Additionally, the combination of using filters and search allow the user to refine the content that is displayed on the screen by category (nutrition, physical activity, obesity) or by keywords.

#### Use Case 2: User Data

**Figure 5:**
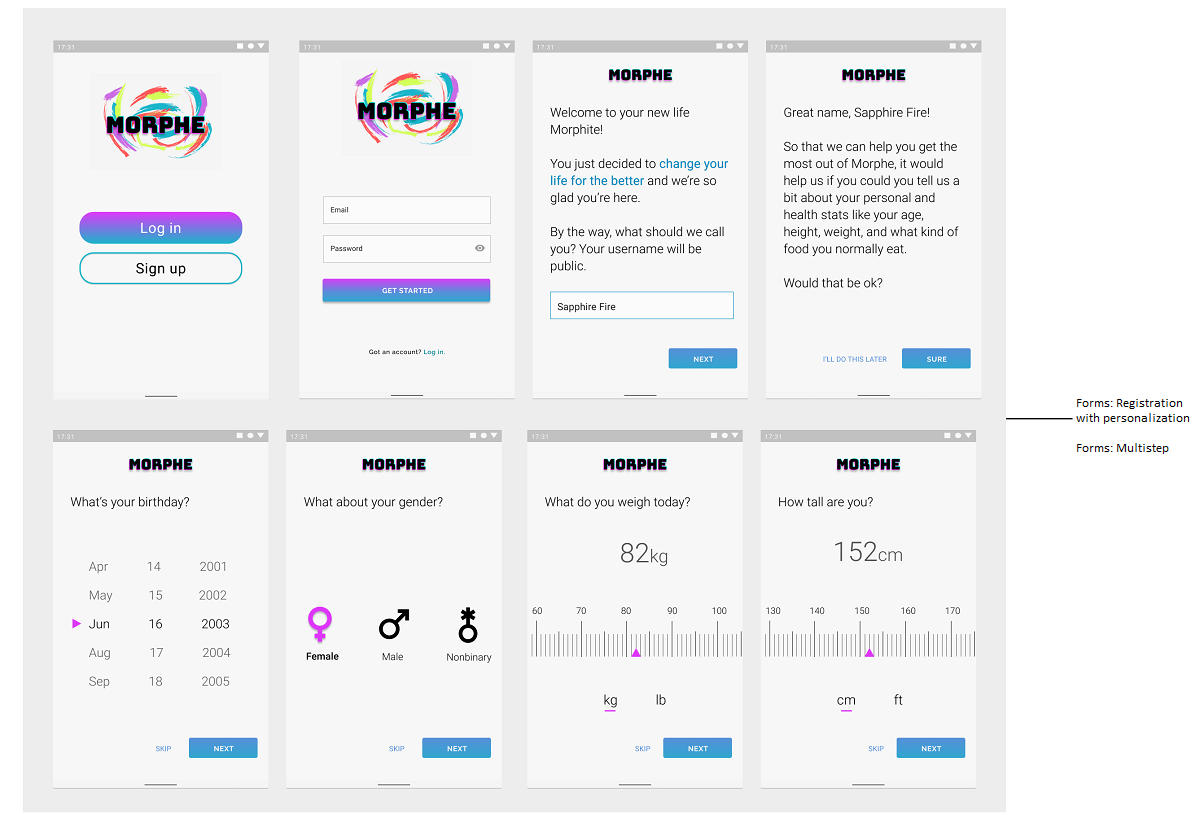
Morphe app registration process.

Forms are used to collect data from the user. These data are primarily used to personalize users’ experiences as assisting with goal setting and providing a baseline for behavioral monitoring. The Morphe app uses a multistep form to gather data about the user in the registration process (Figure 5). The multistep patterns allow each option to be accessible by some other means as well. For example, a BMI calculator that displays a weight range overview dependent on weight and height input can be used to help a user define a goal weight or to see their progress toward a goal weight each time they log in their weight.

#### Use Case 3: Use of Charts

**Figure 6:**
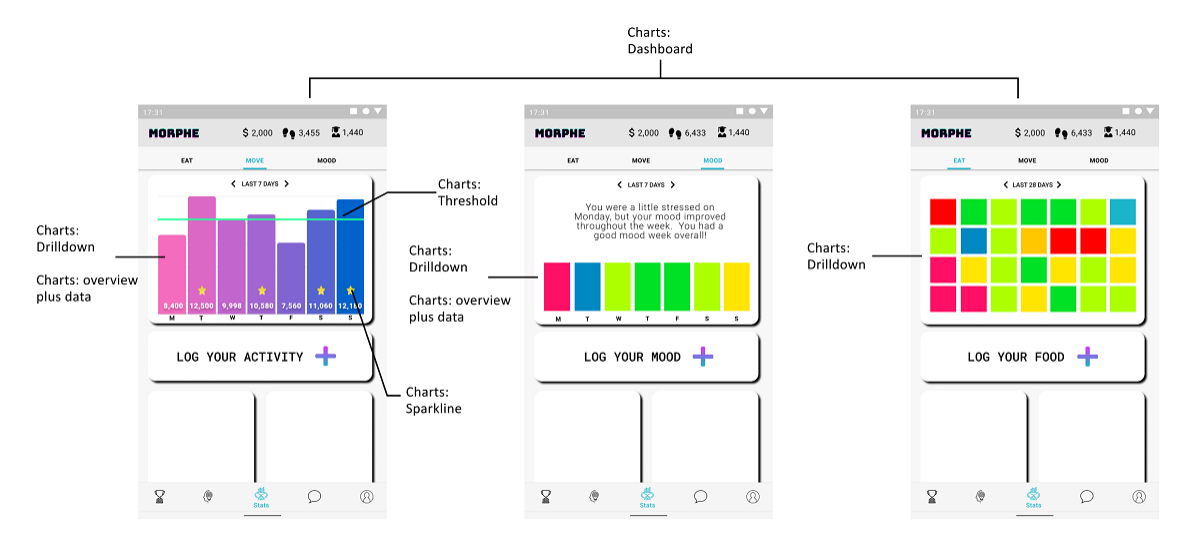
Morphe app statistics.

Charts give the user a way of interacting with their data, including goal and performance history and progress. In the context of behavior change, charts can be used to present a review of the behavioral and outcome goals, represent data from periods in which the user achieved their goals, as well as provide feedback on the user’s performance. In the given example (Figure 6), the statistics functionality provides an overview of the user’s effort and progress concerning the set goals. The stats page includes submenus for diet and nutrition (eat submenu item), exercise (move submenu item), and mental health and well-being (mood submenu item). In order to visualize the progress and achievements, a few types of charts are used: dashboard, drilldown, threshold, sparklines, and overview plus data.

#### Use Case 4: Gamification

**Figure 7:**
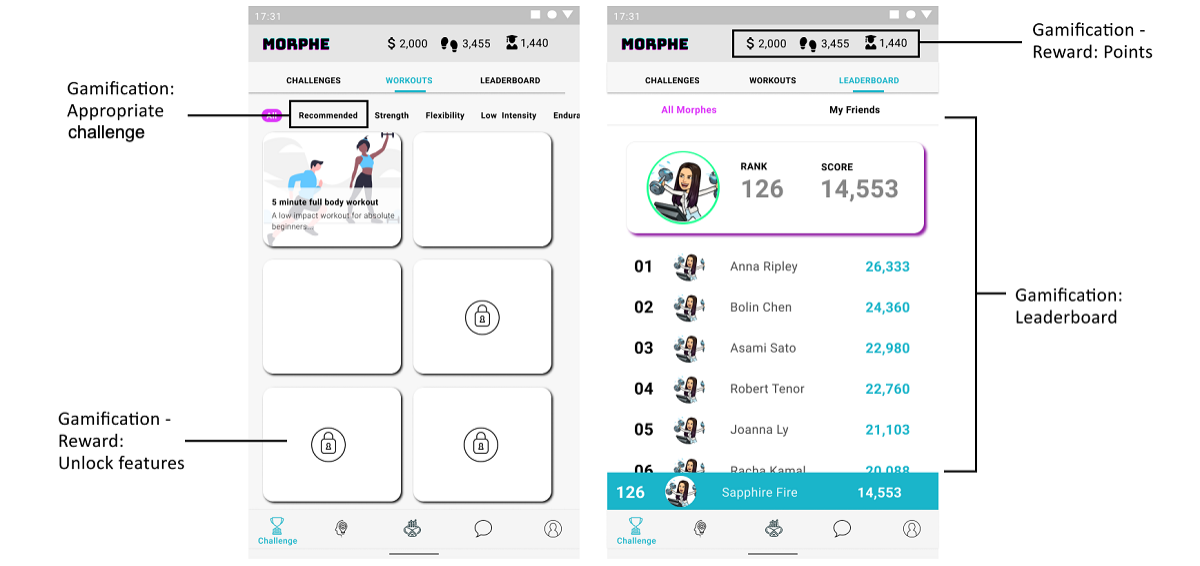
Morphe app challenge.

To engage users in behavior change and achieve their goals, several gamification patterns can be used. The challenge section in the Morphe app (Figure 7) provides access to proposed workouts, challenges, and users’ leaderboards. The workouts submenu allows the user to access workouts and exercises across several categories. Additionally, the user can choose the challenge that is most suitable for achieving a certain goal. As the user progresses throughout the workouts, different features are unlocked as a reward for the effort. Another type of reward is points. The user can be awarded a number of points by winning a physical activity challenge, performing, or making progress toward a goal behavior. In the case of the Morphe app, the points can be used to modify a user’s avatar or “purchase” entry to other challenges. The leaderboard submenu allows the users to view their achievements relative to other users and their friends. This pattern enables the user to compare their achievements with other users, thus identifying their role model and working toward reaching better results.

#### Use Case 5: Notifications

**Figure 8:**
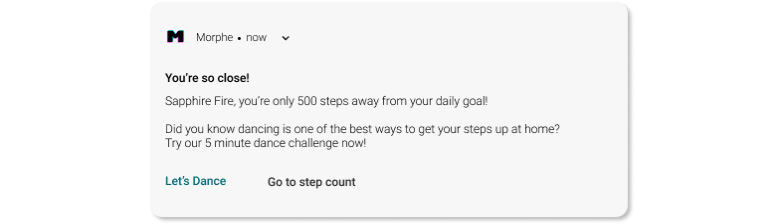
Morphe app notifications.

There are 2 types of notifications: Kairos and Triggers. Kairos are app nudges that utilize personalization and customization patterns to intervene at specific times when the user will be open to receiving advice or performing the goal behavior. For example, the Morphe app uses Kairos (Figure 8) to let the user know when he/she is close to achieving a particular goal and as such, is more likely to feel motivated to give effort toward it.

## Discussion

Behavior change requires highly motivated users; therefore, mHealth apps need to provide features and functionalities that support users’ intrinsic and extrinsic motivation. The apps can increase users’ intrinsic motivation if activities that are interesting, challenging, and that have aesthetic appeal are introduced, while extrinsic motivation can be underpinned if options perceived as valuable, meaningful, and important by the users are presented [23]. Additionally, the expectation for mHealth interventions is to be able to achieve effectiveness in line with the traditional delivery of behavioral interventions. Therefore, the apps developed to support physical activities and healthy eating habits should be engaging enough to motivate the continuation of use.

The proposed design guidelines aim to provide new design considerations by incorporating and supporting BCTs through the use of the recommended UIDPs in the development of mHealth apps. The benefits of UIDPs are to make task completion quicker and easier by reducing cognitive load, thus helping users to achieve behavior change and higher engagement with the apps. The self-regulatory BCTs, including self-monitoring of behavior, goal setting of behavior, and providing feedback on the performance, can be used as feedback processes that are very important in self-management and behavior control [24]. These BCTs have been consistently coupled with positive changes in physical activity [25], and interventions have been found to be more effective when individuals utilize these techniques [26-28]. Moreover, using different patterns from the content, charts, forms, personalization, or gamification categories can increase the successful intervention engagement; therefore, people will engage with mHealth apps in the long term.

Research shows that reward-seeking behavior is more prominent among young people because this age group receives less significant positive responses from rewards, driving them to pursue reinforcers that increase dopamine-related circuitry [29]. As Bryan et al [30] indicate, rewards are experienced in the context of other available rewards, and young people may be particularly sensitive to these changing contexts. Additionally, Davidow et al [31] note that “adolescents are notorious for engaging in reward-seeking behaviors,” and much research in the behavioral health field suggests that the most successful rewards for motivating young people are tied to achieving goals that are immediate, simple, and socioculturally reinforced. This represents an important opportunity to support the users’ extrinsic motivation by diversifying the rewards in the apps by using gamification patterns such as the use of a points system, introducing levels, or offering opportunities to unlock new features. These patterns can provide experiences of autonomy, competence, and relatedness by adding fun and excitement to the activities [32]. Although the implementation of the “Training to use prompts” behavior technique and utilizing the “Onboarding: tutorials” design patterns represent a clear opportunity in future app development, training and navigation menus are important in the early stages of app interaction and adoption. However, the provided instructions need to keep the text to a minimum and written to a sixth-grade level [33]; otherwise, it can be difficult to read, presenting a further barrier to engagement.

The literature regarding the social support for BCTs presents opposite findings. Although some research suggests that young people find peer interactions more rewarding [30], others find that young people find social posting not desirable since they do not want to bother their friends or share achievements that they considered to be uninteresting [34]. Another concern is sharing personal or sensitive data with others [35]. In this context, during the development phase of the app, designs that implement social patterns with relative anonymity that will support the young people’s engagement need to be considered. Stress management BCT has been identified as a necessary component for health behavior change, especially for young people with obesity who might have greater levels of stress [36]. UIDPs included in the content group can be useful in the implementation of this behavior technique by providing generic information about mindfulness, stress, and anxiety management.

BCT categories such as “prompt self-talk” or “prompt identification as a role model” can increase user engagement by focusing on intrinsic and social motivation elements that are recorded as having bolstered engagement [37]. However, effective implementation of prompts needs consideration in timing, frequency, and tailoring [38] that can be designed using Kairos, Triggers, and Personalization in combination with Gamification or Social Patterns. Further, personalized prompts for a given situation are proven to be more effective in behavior change [39]. Problem-solving solutions for barriers to physical activities can motivate individuals to increase their activity [40]. Therefore, features that could assist users with solving barriers to physical activity, such as well-timed notifications for inclement weather or recommendations for suitable in-door exercises can be beneficial. The proposed design guidelines link the BCTs with UIDPs, which can maximize the impact and increase the adoption and continuation of use of mHealth apps for obese young people. However, the design process of such apps is complex and requires the involvement of relevant stakeholders: public health and clinical experts for content creation, app developers for designing the apps’ features and functionality, and young people as prospective users of the app.

A few limitations in this research need to be noted. The time frame of the app analysis should be considered since there is a possibility that some of the features could be revealed to the user after using the app over a longer period of time. The applicability of the proposed BCT-UI design guidelines was presented by designing the prototype app Morphe. However, the prototype was created without insight from the young people community. Furthermore, the guidelines need to be validated with the real users of an mHealth app targeting young people with obesity.

In conclusion, the analysis of 17 mHealth apps has shown that the utilization of UIDPs in implementing BCTs is limited. Taking into account the importance of BCT and UIDP in improving the efficacy of the mHealth apps, in this paper, we proposed BCT-UI design guidelines. The aim of these guidelines is to support the development of mHealth apps that are easy to use and effective for long-term adoption by young people. Additionally, 5 use cases of the Morphe app targeting overweight and obese young people were presented to showcase the usability of the design guidelines. Future research should involve the development and deployment of the Morphe app and validation of its usability and effectiveness in obesity and overweight management within the young people community. However, since the proposed guidelines are generalized, exploring its utilization in the design of mHealth apps for the management of other health conditions as well as various age groups can be valuable.

## Appendix

Multimedia Appendix 1 Coding constraints of the Coventry, Aberdeen, and London-Refined (CALO-RE) taxonomy of behavior change techniques [10].

Multimedia Appendix 2 User interface design pattern definitions [19,21].

Multimedia Appendix 3 Behavior change techniques implemented in the reviewed apps.

Multimedia Appendix 4 Proposed usage of the user interface design patterns in the context of the behavior change techniques.

## Abbreviations

BCT: behavior change technique
BCT-UI: behavior change technique–user interface
CALO-RE: Coventry, Aberdeen, and London-Refined
mHealth: mobile health
UIDP: user interface design pattern
