# Supplementary material for: mHealth Apps Targeting Obesity and Overweight in Young People: App Review and Analysis
Source: JMIR Mhealth Uhealth. 2023 Jan 19;11:e37716. doi: 10.2196/37716 (PMC9896356; doi:10.2196/37716)
Supplement: Multimedia Appendix 1 [file mhealth_v11i1e37716_app1.pdf]

Multimedia Appendix 1: Coding constraints for the refined taxonomy of BCT - Coventry, Aberdeen, and London-Refined (CALO-RE) [10]

#	CALO-RE
1	Information provision (general): Coded wherever generic positive information about physical activity or nutrition was provided.
2	Information provision (to the individual): Coded wherever specific information about the possible outcomes of engaging in the desired behavior were mentioned in the context of the user or a relevant group based on the user's characteristics.
3	Information provision (others' approval): Coded wherever information about others' perceptions of the user was provided.
4	Information provision (others' behavior): Coded wherever a normative user's activities with respect to physical activity was provided. Not about specific users.
5	Goal setting (behavior): Coded wherever goals to begin or maintain behavior change, were set. Goals may be non-specific (e.g. Exercise more).
6	Goal setting (outcome): Coded wherever a user is encouraged to set general goals achievable through performance of the behavior (e.g. Lose weight).
7	Action planning: Coded where detailed plans are made to act including when and where. Not coded if a plan does not link to a specific situational cue (if-then).
8	Identifying barriers/problem resolution: Coded wherever user was asked to identify barriers to performing the behavior and possible solutions.
9	Setting graded tasks: Coded wherever a target behavior is broken into smaller, easier to achieve tasks, but progressively harder tasks.
10	Review of behavioral goals: Coded wherever the user is given an opportunity to review accomplishment of previous goals, including opportunities to modify the action plan.
11	Review of outcome goals: Coded wherever a review or analysis of an outcome goal is provided.
12	Effort or progress contingent rewards: Coded wherever rewards or praise are given for attempts at achieving the goal behavior.
13	Successful behavior contingent rewards: Coded wherever rewards or praise are given for successful performance of the goal behavior.
14	Shaping: Coded wherever rewards are given for progress towards completion of a target behavior. Rewards are graded.
15	Generalization of target behavior: Coded wherever users are encouraged to find opportunities to perform the goal behavior in alternate situations.
16	Self-monitoring of behavior: Coded wherever a user can record activities performed in line with attempting or achieving the goal behavior (e.g. Duration or distance of run).
17	Self-monitoring of behavioral outcome: Coded wherever a user can record outcomes of performed behavior (e.g. Weight change).
18	Focus on past success: Coded wherever a user is encouraged to reflect on successful past experiences of the goal behavior.

19	Provide feedback on performance: Coded wherever the user is provided with informative or evaluative feedback on the performance of the behavior (e.g. form, frequency, duration, intensity).
20	Informing when and where to perform behavior: Coded where suggestions or advice as to time and location for performance of the goal behavior are provided.
21	Instruction on how to perform behavior: Coded where instructions are provided as to how to perform the goal behavior.
22	Demonstrate behavior: Coded where a demonstration of the goal behavior is provided.
23	Training to use prompts: Coded where instructions are provided as to how to use cues/reminders for the performance of goal behavior.
24	Environmental restructuring: Coded where the user is encouraged to change their environment to facilitate the performance of the goal behavior.
25	Agreement of behavioral contract: Coded where the user is asked to 'sign' a 'contract' that records an agreement to perform the goal behavior.
26	Prompt practice: Coded where the user is reminded to rehearse and repeat the behavior or situations that lead to the behavior.
27	Use of follow-up prompts: Coded where the user is reminded to perform the behavior in longer time intervals after the achievement of a behavior.
28	Facilitate social comparison: Coded where users are encouraged to draw comparisons with others' behavior.
29	Plan social support: Coded where social groups are used to facilitate completion of the behavior.
30	Prompt identification as a role model: Coded where users are encouraged to act as a role model (e.g. Urging friends and family to engage in physical activity).
31	Prompt anticipated regret: Coded where anticipated negative feelings associated with failure to perform the behavior are highlighted.
32	Fear arousal: Coded where fear-inducing information about the consequences of failure to achieve the goal outcome is presented to the user.
33	Prompt self-talk: Coded where the user is encouraged to offer themselves verbal encouragement to perform the behavior.
34	Prompt use of imagery: Coded where the user is encouraged to imagine themselves successfully performing the goal behavior.
35	Relapse prevention: Coded where the user is encouraged to make plans to maintain the behavior after the goal is reached.
36	Stress management: Coded where encouragement or techniques to reduce stress and improve emotional control are provided.
37	Motivational interviewing: Unable to code due to restrictions in CALO-RE instructions.
38	Time management: Unable to code due to restrictions in CALO-RE instructions.
39	General communication skills training: Unable to code due to restrictions in CALO-RE instructions.

40	Stimulate anticipation of future rewards: Code this technique when participants are told at the onset that they will be rewarded based on behavioral achievement.
