# Supplementary material for: mHealth Apps Targeting Obesity and Overweight in Young People: App Review and Analysis
Source: JMIR Mhealth Uhealth. 2023 Jan 19;11:e37716. doi: 10.2196/37716 (PMC9896356; doi:10.2196/37716)
Supplement: Multimedia Appendix 2 [file mhealth_v11i1e37716_app2.pdf]

## Multimedia Appendix 2: User interface design patterns definitions [19, 21]

| UIDP | |
|---|---|
| **Charts** | *Dashboard*<br><br>Provide several different data visualisations together on screen. These are usually navigable / drilldown charts. |
| | *Drilldown*<br><br>Invite the user to Drill Down for more data. Use the OS-appropriate control for navigating back up the chain (i.e., the Back button for iOS and the Up button for Android). |
| | *Interactive Preview*<br><br>Provide a chart or data visualisation that changes in response to user input. |
| | *Overview plus data*<br><br>Use this pattern where a visual overview accompanying the detailed data will be more valuable to your users than just a list of transactions with no summary. |
| | *Sparklines*<br><br>Sparklines, also called micro charts, are small intense, simple, word-sized graphics. Consider using Sparklines as the entry point to a Drill Down into a more detailed view. |
| | *Threshold*<br><br>Use colours, bands, or lines on charts to indicate threshold information about the data. |
| **Content** | *Article List*<br><br>Provide informational article teasers in a scrollable list. Each item element should contain a title, a short description, a date, and a call to action that encourages clicking on the item. |
| | *Cards*<br><br>Display entry points to detailed and varied content in similarly sized rounded rectangles with a slight drop shadow. A card could contain a photo, text, and a link about a single subject. |
| | *Favorites*<br><br>Provide a favorite button, juxtaposed to items in a list view or placed within a detail view. The button usually takes the shape of a star or heart, though may sometimes be implemented as a bookmark icon, and called "save". Additionally, consider providing a list of items that the user has favored. |
| | *Filter*<br><br>Provide a way to refine data sets using some criteria to return only results deemed relevant by the user. Filters can be onscreen, part of a dropdown list, or displayed as an overlay upon button click. |

| | |
|---|---|
| | *Search*<br>Implement a search bar wherever a user needs to search content. Search bars should include recent or saved searches to minimize the need for typing. |
| | *Social Proof*<br>Highlight the activity you want the user to conduct by framing it socially. (100 people like you completed this challenge today!) |
| **Forms** | *Calculator*<br>A form contains input fields that allow a user to obtain a result. The result can be visual in the form of a chart or graphic, or it can be a text or numerical result. Calculator forms work best in conjunction with an interactive preview. |
| | *Multistep*<br>A form is broken into multiple steps over several pages. |
| | *Registration with Personalization*<br>Collect information that will personalize the experience of the app to the user during registration. |
| **Gamification - Reward** | *Collectibles*<br>Collectibles are achievements that can be collected by the user. |
| | *Points*<br>Incorporate a points system, where you receive points for achieving a goal or participating in a behavior. Points can be in the form of "coins" or some other barterable item that can be used to customize your experience. |
| | *Praise*<br>Use explicit statements, graphics, a sound effect, or a similar indicator to reward a user. |
| | *Unlock features*<br>Only allow users to experience certain features as a reward for a specific behavior. |
| **Gamification** | *Appropriate Challenge*<br>To keep users in flow we need to give them Appropriate challenges. If a challenge is too hard, the user is going to feel stress and anxiety. If the challenge is too easy, the user is going to feel bored. Both boredom and anxiety tend to lead to disengagement from the activity that was previously rewarding. |
| | *Leaderboard*<br>Provide a way for users to see how they are doing when compared to their peers in the context of a specific challenge or goal behavior. |
| | *Levels* |

| | |
|---|---|
| | Incorporate a "level" system that partitions challenges or goals into different levels of difficulty, with rewards in increasing levels of desirability. |
| **Notification** | *Kairos*<br><br>Kairos is “a passing instant when an opening appears which must be driven through with force if success is to be achieved”. Kairos are like triggers that should be implemented in situations of change, i.e. in response to some situation or event that represents a moment when users are open and receptive to engaging in behavior change. Kairos need to be learned over time. |
| | *Trigger*<br><br>Use small nudges to cue users to act – directly or through learned associations. |
| **Onboarding** | *Tutorials*<br><br>Offer users a tutorial that teaches them, preferably interactively, about the features of the app. |
| **Personalization** | The system adapts its interface to the perceived need of a user |
| **Customization** | The user can make changes to the interface or content received directly to tailor their own experience. |
| **Scarcity** | Indicate that a benefit or reward is only available in limited number or for some limited time. |
| **Social** | *Activity Stream*<br><br>A series of broadcasts or notifications that illustrate recent events. |
| | *Comments*<br><br>Provide a user interface element that allows a user to post a text comment in response to another user's activity or post. |
| | *Connecting*<br><br>Offer connection points with existing social media sites. |
| | *Groups*<br><br>Offer the ability to join or create groups, which act as communities for social connection and sharing. |
| | *Reactions*<br><br>React to an activity or post in a positive manner. |
| | *Profile*<br><br>Provide a user interface component where a user can present an image of themselves to other users. Usually inclusive of a profile picture or avatar, a bio or user interests, and possibly their activity. |
