# Supplementary material for: mHealth Apps Targeting Obesity and Overweight in Young People: App Review and Analysis
Source: JMIR Mhealth Uhealth. 2023 Jan 19;11:e37716. doi: 10.2196/37716 (PMC9896356; doi:10.2196/37716)
Supplement: Multimedia Appendix 3 [file mhealth_v11i1e37716_app3.pdf]

Multimedia Appendix 3: Behavior change techniques implemented in the reviewed apps

	#1 - Information provision (general)	#3 - Information provision (others' approval)	#4 - Information provision (others' behavior)	#5 - Goal setting (behavior)	#6 - Goal setting (outcome)	#7 - Action planning	#9 - Setting graded tasks	#12 - Effort or progress contingent rewards	#13 - Successful behavior contingent rewards	#14 - Shaping	#16 - Self-monitoring of behavior	#17 - Self-monitoring of behavioral outcome	#19 - Provide feedback on performance	#21 - Instruction on how to perform behavior	#22 - Demonstrate behavior	#26 - Prompt practice	#28 - Facilitate social comparison	#29 - Plan social support	#36 - Stress management	#40 - Stimulate anticipation of future rewards
NFL Play 60	√						√	√	√	√										
Runtastic (Adidas Running)	√	√		√							√						√			
7-Minute Workout									√		√	√	√		√	√				
Sworkit							√				√			√	√	√				
Couch to 5K (C25K)							√				√			√		√				
Endomondo				√				√			√		√				√	√		
Fitify									√	√	√		√	√	√	√				
Fitness Buddy				√	√						√	√	√	√	√					
FitOn	√		√	√	√	√			√		√			√	√	√	√	√		√
FitBit	√		√	√	√			√	√		√	√	√	√	√		√	√	√	√
Zombies, Run!							√		√	√			√	√						√
Walkr							√		√		√					√	√			
Cron-O-Meter				√	√						√	√	√							
Eat the Rainbow Food Journal	√			√				√		√	√									√
MyFitnessPal	√		√	√	√						√	√	√			√				
Lose It! Calorie Counter					√				√		√	√	√			√	√	√		
Fitatu					√						√	√				√				
