# Supplementary material for: mHealth Apps Targeting Obesity and Overweight in Young People: App Review and Analysis
Source: JMIR Mhealth Uhealth. 2023 Jan 19;11:e37716. doi: 10.2196/37716 (PMC9896356; doi:10.2196/37716)
Supplement: Multimedia Appendix 4 [file mhealth_v11i1e37716_app4.pdf]

Multimedia Appendix 4: Proposed usage of the user interface design patterns in the context of the behavior change techniques

UIDP	Example Usage
**Charts: Dashboard**	*Review of behavioral Goals:* Use a dashboard view to display the user's average daily step count, caloric intake, and number of workouts over a given week to tell a story about whether the user's behavioral goals have been achieved and whether they need to be reviewed<br>*Review of Outcome Goals:* Use a dashboard view to display the user's weight change, BMI change, and body fat percentage changes over time to tell a story about whether the user's outcome goals have been achieved and whether they need to be reviewed<br>*Focus on past success:* Use a dashboard view to display any combination of charts that represent data from time periods in which the user achieved their goals.<br>*Provide feedback on performance:* Use a dashboard view to display the user's average daily step count, caloric intake, and number of workouts over a given week to tell a story about how the user is progressing toward their behavioral goals
**Charts: Drilldown**	*Provide feedback on performance:* A progress indicator may be displayed on the user's dashboard. Clicking on the progress indicator will give more details about specific aspects of that progress such as step count this week, days active etc.
**Charts: Interactive Preview**	*Goal setting (outcome):* A BMI calculator that displays a weight range overview dependent on weight and height input can be used to help a user define a goal weight.<br>*Action planning:* A sparkline may indicate respective impact on a user's goals of actions planned if they are taken, as actions are set.<br>*Review of outcome goals:* A BMI calculator that displays a weight range overview dependent on a user's current weight and height input can be used to help a user redefine a goal weight.<br>*Self-monitoring of behavior:* A sparkline may be used to indicate a user's progress toward their weekly physical activity goals. The sparkline is updated as the user inputs more activity.<br>*Self-monitoring of behavioral outcome:* A line graph may be used to indicate a user's progress toward their weight loss goals. The line graph is updated as the user logs their weight.<br>*Relapse prevention:* In planning behavior maintenance the user may be presented with a chart that displays their performance given their current goals. The chart will change as the user modifies their goals until it displays a suitable option for the user.

**Charts: Overview plus data**	*Review of behavioral goals, Review of outcome goals, Focus on past success, Provide feedback on performance:* A line graph shows a user's physical activity over a time. Below the graph is a list of activities engaged in over that time. If 'Focus on past success' the graph should represent a time period whether the user successfully achieved the desired behavior.
**Charts: Sparklines**	*Setting graded tasks:* A progress bar can infer that tasks are graded. The bar may change colour as it progressed towards a goal to indicate the effort required to complete the next task.<br><br>*Focus on past success:* Small graphical representations of behavior can indicate the user's previous achievements<br><br>*Provide feedback on performance:* A coloured bar can show progress towards an activity goal such as steps count.<br><br>NB. Sparklines are often used as the entry point for Drilldown Charts
**Charts: Threshold**	*Review of behavioral Goals:* Use a bar chart to display workouts performed over a given week, with differently coloured bars to indicates which days represent a successful achievement of a behavioral goal to tell a story about whether the user's behavioral goals have been achieved and whether they need to be reviewed<br><br>*Review of Outcome Goals:* Use a line chart with a benchmark that indicates the user's goal weight to display the user's weight change over time to tell a story about whether the user's outcome goals have been achieved and whether they need to be reviewed<br><br>*Focus on past success:* A bar chart that represents daily step count over time may indicate by way of colours or lines the days on which the user achieved their step count behavioral goal.<br><br>*Provide feedback on performance:* A bar chart that displays daily calorie intake may have a line through it to indicate a threshold that represents a user's progress towards a goal caloric intake.

**Content: Article List**	Providing a list articles for the user to scan and consume. Article lists tend to contain multiple rectangular cards that span the width of the screen, with each card containing an image, a title and a brief description to allow the user to understand what information is contained in the article.<br><br>Information provision (general), Information provision (to the individual), Information Provision (others' behavior), Informing when and where to perform behavior, Instruction on how to perform behavior, Fear arousal: Articles can provide general information or be used to inform a user about specific behaviors and their consequences of non-performance both generally, and with specific view of the user or their normative group.<br><br>*Identifying barriers/problem resolution:* Articles can offer solutions for common barriers to behavior change.<br><br>*Environmental restructuring, Generalization of target behavior:* Articles can contain encouragement to perform a target behavior in alternate situations, or to change elements of the user's environment to facilitate performance of the behavior.<br><br>*Focus on past success:* Article lists can be combined with sparklines and used as the entry point for drilldown charts to display a list of successes.<br><br>*Relapse Prevention:* Articles can offer options for continuation of the behavior after goals have been reached.<br><br>*Stress Management:* Articles can contain advice to improve a user's mental health by providing stress and anxiety management techniques for the user to engage in.<br><br>NB: It would make sense to implement an article list with the following patterns: cards, favorites, search, filter.
**Content: Cards**	Cards are variable sized rectangular containers used to store and deliver content. They can be used in a number of ways - to display article content for an article list, to hold charts, to hold challenge description or behavior instruction, or as a view for user achievements.
**Content: Favorites**	Many items can be favorited, from articles, to exercises, to challenges. Favoriting can be implemented as a "save" or "heart" on an item. Favorited items should be accessible easily.
**Content: Filter**	Filters allow a user to refine the content that is displayed on the screen. They can be implemented in combination with most other patterns. An article list may be filtered by category, challenges may be filtered by type (nutrition or physical activity), data overview may be filtered by some time period.
**Content: Search**	A search bar allows the user to locate content of interest to them by inputting relevant keywords. Search can be implemented in combination with any other content pattern.

**Content:**<br><br>**Social Proof**	Textual references to community engagement:<br><br>"300 people who share your weight goal accepted this challenge today"<br><br>"1300 users achieved their step goal today, you can too!"<br><br><br>There are many opportunities for use of social proof. This could be incorporated into any instance we would like to encourage the goal behavior, whether it be part of a trigger to engage in the behavior, or within the description of a challenge.
**Forms: Calculator**	*Goal setting (outcome), Review of Outcome Goals, self-monitoring of behavioral outcome:* A BMI calculator that displays a weight range overview dependent on weight and height input can be used to help a user define a goal weight, or to see their progress towards a goal weight each time they log their weight.<br><br>*Review of behavioral Goals, Self-Monitoring of behavior:* A daily calorie intake goal might be calculated from a user's goal weight during review, or calories remaining might be calculated each time the user inputs their nutrition intake.
**Forms: Multistep**	Multistep forms, similar to cards, are containers for data input. Anywhere the system requires data input from the user, the designer should consider implementing this process as a multi-step form.
**Forms: Registration with Personalization**	Ask the user about their height, weight, and goals during the registration process.<br><br>Ask the user to plan when and where they will perform the activity during registration.<br><br>Ask the user to identify deterrents to performing the activity and list possible solutions during registration.<br><br>Ask the user to indicate their contractual agreement to engage in the desirable behavior during registration.<br><br>Ask the user to set up prompts that suit them during the registration process.<br><br><br>Note: Registration should not be an excessively lengthy process as this can cause users to give up on app usage. Each option should be accessible by some other means as well.
**Gamification - Reward: Collectibles**	*Effort or progress contingent rewards, Successful behavior contingent rewards, Shaping:*<br><br>Collectibles can be implemented as badges or awards that can be awarded based on performance of the goal behavior, achievement towards goal outcome, winning a challenge or completing a challenge. Collectibles can become harder to achieve over time to support Shaping. Grouping collectibles into families makes use of the set completion pattern as well.

**Gamification - Reward: Points**	Effort or progress contingent rewards, Successful behavior contingent rewards, Shaping:<br>Winning a physical activity challenge, performing a gaol behavior, or making progress towards a goal behavior rewards a user with some number of coins. Coins can then be used to modify a user's avatar, or "purchase" entry to other challenges. The harder the activity engaged in, the more coins the user can win to support Shaping.
**Gamification - Reward: Praise**	The app notifies the user with a pleasant tone and text alert when they achieve or make progress towards a goal.
**Gamification - Reward: Unlock features**	*Effort or progress contingent rewards, Successful behavior contingent rewards, Shaping:*<br>A user may not be able to participate in certain challenges that offer greater rewards unless they have unlocked this feature by winning or participating in some number of challenges.<br>A user may not be able to interact with other users by commenting, liking, or messaging them unless they have unlocked this feature by creating a behavioral contract.
**Gamification: Appropriate Challenge**	We may use input from the user about their current activity level, their current nutrition status, and their feelings towards healthy eating and exercise to recommend appropriate challenges, exercises, and goals.
**Gamification: Leaderboard**	*Prompt identification as a role model, Facilitate social comparison:* A list of users that have completed a challenge such as a daily step count challenge sorted by achievement (number of steps). Top users may be highlighted for 'Prompt identification as a role model'.
**Gamification: Levels**	*Shaping, Setting graded tasks:*<br>By completing challenges, a user's "level" rises. Challenges become more difficult, meaning it is harder and harder to achieve the next level.
**Notification: Kairos**	Kairos are nudges from the system that are implemented at "opportune times for behavior change". They utilize the cur and customization patterns to intervene at specific times when the user will be open to receiving advice or performing the goal behavior.<br>An example for Kairos may present itself by making use of geotracking. An app could ask you to drop pins in "locations of temptation" - like a fast food restaurant. When the app recognises that the user is geographically close to a fast food restaurant, for instance, it could be triggered to send the user an encouraging text message that may suggest an alternative, healthier, eatery. We could also use Kairos to let the user know when they are close to achieving their goal and as such more likely to feel motivated to do a little more towards it.
**Notification: Trigger**	Triggers can be set - such as an alert or reminder to exercise or complete a challenge, or they can come in the form of distractions such as notifications, text messages or overlays. Any content, reminder or encouragement can come in the form of a trigger.

**Onboarding: Tutorials**	On first use, a user is greeting with a playthrough, whereby the app teaches them about the features of the app by having the user complete certain tasks and navigate to different pages in the app.
**Personalization**	The app may use information it learns about the user's physical activity to suggest appropriate challenges or goals. It may also learn through the user's content "likes", which articles the user prefers to read and may tailor their view as such.
**Customization**	An exercise routine offers the user the opportunity to modify the length of the workout, how many breaks they would like to take, and the accent of the virtual coach.
**Scarcity**	*Effort or progress contingent rewards, Successful behavior contingent rewards, Shaping, Stimulate anticipation of future rewards:* Scarcity can be implemented in combination with other patterns such as Kairos, Triggers, or cards, can be visually represented using sparklines, or simply be textual. Examples include indicating that only the first XX number of participants in a challenge can receive a coveted reward.
**Social: Activity Stream**	An activity stream displays recent activity and posts from other users.
**Social: Comments**	An input box allows a user to comment on another user's post or activity.
**Social: Connecting**	Offer integration with a social media account, at sign up or using various connection points throughout the app.
**Social: Groups**	Include a group creation feature that allows users to connect with like-minded individuals. Within a group component, users should be able to undertake conversations and interact with each other through comments, messages, and reactions.
**Social: Reactions**	Reactions can be incorporated as likes (thumbs up), loves (hearts), or similar, on a post in another user's activity stream.
**Social: Profile**	A profile page for an application's user introduces this user with a photo or avatar, short bio, and lists of interests. This is complemented by the activity stream pattern. The profile might also display the user's “reach” by way of points, levels, or achievements, and offer a way to connect with them.
